# Cardiac Adaptation to Prolonged High Altitude Migration Assessed by Speckle Tracking Echocardiography

**DOI:** 10.3389/fcvm.2022.856749

**Published:** 2022-05-23

**Authors:** Xu Chen, Bohan Liu, Yujiao Deng, Feifei Yang, Wenjun Wang, Xixiang Lin, Liheng Yu, Haitao Pu, Peifang Zhang, Zongren Li, Qin Zhong, Qian Jia, Yao Li, Xiao Wang, Wei Chen, Daniel Burkhoff, Kunlun He

**Affiliations:** ^1^Beijing Key Laboratory for Precision Medicine of Chronic Heart Failure, Key Laboratory of Ministry of Industry and Information Technology of Biomedical Engineering and Translational Medicine, Translational Medicine Research Center, Medical Artificial Intelligence Research Center, Chinese PLA General Hospital, Beijing, China; ^2^BioMind Technology, Zhongguancun Medical Engineering Center, Beijing, China; ^3^Department of Ultrasound Diagnosis, The Seventh Medical Center of Chinese PLA General Hospital, Beijing, China; ^4^Cardiovascular Research Foundation, New York, NY, United States

**Keywords:** high altitude acclimatization, cardiac responses, cardiac physiology, echocardiography, strain

## Abstract

**Objective:**

Exposure to high altitudes represents physiological stress that leads to significant changes in cardiovascular properties. However, long-term cardiovascular adaptions to high altitude migration of lowlanders have not been described. Accordingly, we measured changes in cardiovascular properties following prolonged hypoxic exposure in acclimatized Han migrants and Tibetans.

**Methods:**

Echocardiographic features of recently adapted Han migrant (3–12 months, *n* = 64) and highly adapted Han migrant (5–10 years, *n* = 71) residence in Tibet (4,300 m) using speckle tracking echocardiography were compared to those of age-matched native Tibetans (*n* = 75) and Han lowlanders living at 1,400 m (*n* = 60).

**Results:**

Short-term acclimatized migrants showed increased estimated pulmonary artery systolic pressure (PASP) (32.6 ± 5.1 mmHg vs. 21.1 ± 4.2 mmHg, *p* < 0.05), enlarged right ventricles (RVs), and decreased fractional area change (FAC) with decreased RV longitudinal strain (−20 ± 2.8% vs. −25.5 ± 3.9%, *p* < 0.05). While left ventricular ejection fraction (LVEF) was preserved, LV diameter (41.7 ± 3.1 mm vs. 49.7 ± 4.8 mm, *p* < 0.05) and LV longitudinal strain (−18.8 ± 3.2% vs. −22.9 ± 3.3%, *p* < 0.05) decreased. Compared with recent migrants, longer-term migrants had recovered RV structure and functions with slightly improved RV and LV longitudinal strain, though still lower than lowlander controls; LV size remained small with increased mass index (68.3 ± 12.7 vs. 59.3 ± 9.6, *p* < 0.05). In contrast, native Tibetans had slightly increased PASP (26.1 ± 3.4 mmHg vs. 21.1 ± 4.2 mmHg, *p* < 0.05) with minimally altered cardiac deformation compared to lowlanders.

**Conclusion:**

Right ventricular systolic function is impaired in recent (<1 year) migrants to high altitudes but improved during the long-term dwelling. LV remodeling persists in long-term migrants (>5 years) but without impairment of LV systolic or diastolic function. In contrast, cardiac size, structure, and function of native Tibetans are more similar to those of lowland dwelling Hans.

## Introduction

High altitude exposure is associated with pulmonary hypertension and pulmonary vasoconstriction resulting in significant changes in cardiovascular properties (1). There are many factors that affect cardiac adaptation to high altitude dwelling, including altitude level, exposure time, and racial differences. Cardiac responses to acute hypoxic exposure in people normally living at low altitudes (lowlanders) have been studied extensively (2, 3). Conventional echocardiographic studies show that in healthy subjects exposed to acute hypoxic conditions of high altitude, the systolic function of both ventricles is preserved, but there is impaired diastolic function (4). More recently, speckle tracking echocardiography (STE) has been introduced, which has better reproducibility and sensitivity than conventional echocardiography for quantifying cardiac functions through analysis of deformation mechanics (5). The use of STE is more sensitive for exploring subclinical changes in cardiac function to high altitude dwellings. Maufrais et al. (6) noted that after several days (<6 days) at high altitude, there are no significant changes in left ventricular (LV) or right ventricular (RV) longitudinal strain, but there is increased LV twist. In contrast, Stembridge et al. (7) reported that RV longitudinal strain is decreased, which is a consequence of high pulmonary artery systolic pressure (PASP) following longer duration trekking (>10 days).

However, there is limited information concerning how healthy lowlanders (i.e., individuals who themselves and their ancestors are born and living below 2,500 m) adapt to longer-term (months-to-years) exposure to high altitudes (greater than 2,500 m); in particular, there are no data concerning changes in cardiac mechanics under such conditions. An example is when healthy Han individuals (the most common race in China) born and living at low altitudes (<2,500 m) migrate to the Tibet plateau (>4,000 m). It is not uncommon for young adults to live and work at high altitudes over periods of months to years, which provides the opportunity to study long-term cardiovascular adaptations to chronic hypoxia in these extreme conditions.

Accordingly, the purpose of this study was to provide a comprehensive characterization of changes in cardiac structure, function, and longitudinal strain during prolonged (month-to-years) exposure to high altitude (4,300 m) in Han migrants and compare them to characteristics of native Tibetans. We hypothesized that (1) cardiac structure (indexed by LV and RV size and wall thicknesses) and function [indexed by LV ejection fraction (EF), RV fractional area change (FAC), and global longitudinal strain (GLS)] of long-term (>5 years) Han migrants are more highly adapted to high altitude than recent acclimatized Hans (<1 year) and (2) despite long-term dwelling (>5 years), these same indexes of cardiac structure and function of Han migrants are not as highly adapted to high altitude as native Tibetans.

## Methods

### Study Population

Han lowlanders were recruited in Yecheng, Xinjiang (1,400 m); Han migrants and Tibetans were recruited in the Ali district of Tibet (4,300 m). A total of 328 volunteers completed a questionnaire and were given free physical and echocardiographic examinations. The questionnaire included age, ethnicity, duration of residence, family medical history, questions related to physical and psychological discomfort, and medications. As the aim of the study was to investigate the normal process of cardiac adaption to chronic hypoxia of young and healthy migrants from lowland, the criteria for being included in this study were as follows: age between 18 and 32 years, male, and a normal physical examination. Exclusion criteria included any history of smoking, self-reported history of any cardiorespiratory disease, obesity, or intake of any medication within a month before participation in this study. After excluding 36 subjects due to insufficient image quality, 292 participants were included in this study, including 217 Hans and 75 Tibetans. Participants enrolled were grouped according to ethnicity, altitude, and adaptation periods into one of 4 groups: (1) Han lowlanders (*n* = 82); (2) short-term acclimatized Hans (*n* = 64), who were defined as Han migrants with residence time in Tibet of more than 3 months but less than 1 year; (3) long-term acclimatized Hans (*n* = 71), who were defined as Han migrants with residence time in Tibet for more than 5 years; and (4) native Tibetans (*n* = 75). The protocol was approved by the Chinese PLA General Hospital Ethics Committee, and each participant provided written informed consent.

### Basic and Clinical Data Acquisition

Data were collected by two physicians and an experienced sonographer who traveled between Yecheng and Ali. Measurement included height, weight, blood pressure (BP), heart rate (HR), arterial O_2_ saturation (SpO_2_), and blood samples. BP and HR were measured (HEM-7211; OMRON, Japan) in the supine position after 10 min of rest. SpO_2_ was measured using finger-pulse oximetry (YX303, Yuwell, Jiangsu, China) after finger warming and signal stabilization. Hemoglobin concentration (HB) and hematocrit (HCT) measurements (BC-3000Plus, Mindray, Shenzhen, China) were performed by peripheral venous sampling. From these data, body surface area (BSA) and body mass index (BMI) were calculated.

### Echocardiographic Assessments

Echocardiographic image acquisition included 2D and Doppler echocardiography and was saved in the DICOM format. The procedure was performed by a certified sonographer using a portable ultrasound machine (M9, probe SP5-1; Mindray, Shenzhen, China) in the left lateral decubitus position according to society guideline recommendations (8, 9). Measurements were performed offline by an experienced physician blinded to the subjects' conditions and ethnicity. LV end-diastolic volume (EDV) and end-systolic volume (ESV) were measured from planar tracings of the endocardial border in the apical 4- and 2-chamber (4C and 2C, respectively) views using the Simpson's biplane method. LV stroke volume (SV) and EF were calculated as EDV-ESV and (EDV-ESV)/EDV, respectively. LV mass (in g) was calculated according to the cube formula using end-diastolic values of septal and posterior wall thickness (LVST and LVPWT) and the LV diastolic dimension (LVDD) from M mode tracings:


$$\begin{array}{l} \text{LVmass }=\;0.8\;\cdot \;1.04\;\cdot \;[(\text{LVDD }+\text{ LVPWT }+\text{ IVST})3\\ \;-{\text{LVDD}}^{3}]\;+\;0.6 \end{array}$$

Right ventricular end-diastolic and end-systolic areas (EDA and ESA) were measured from planar tracings of the endocardial border in the apical 4C view, and RV FAC was calculated by (EDA-ESA)/EDA. Right and left atrial EDVs and ESVs were determined from planar tracings of the endocardial border in the apical 4C view by using the Simpson's method; atrial emptying fractions were calculated by (EDV-ESV)/EDV. Finally, volumes and dimensions were indexed to BSA to balance differences in body sizes. Early (E) and late (A) diastolic filling velocities were assessed using pulsed-wave Doppler recordings, and free wall velocities (s′, e′, and a′) were measured using tissue Doppler imaging at both mitral and tricuspid annular levels. Isovolumic relaxation time (IVRT) and deceleration time (DT) were measured at the level of the mitral annulus. Tricuspid annular plane systolic excursion (TAPSE) was measured from M-mode tracings. Systolic tricuspid regurgitation (TR) flow velocity was used to estimate pressure gradient (*P*_TR_, in mmHg) from the modified Bernoulli equation (10): *P*_TR_ = 4·Vmax^2^. Right atrial pressure (RAP) was estimated by the diameter of the inferior vena cava and its variation during inspiration as detailed previously (11). PASP was calculated as PASP = *P*_TR_ + RAP. Mean PAP (PAMP) was calculated from PASP as PAMP = 0.61 PASP + 2 mmHg (12). The TAPSE/PASP ratio, a surrogate of the gold standard ratio of end-systolic to arterial elastance (Ees/ Ea), was calculated to evaluate RV-arterial coupling (13).

The longitudinal strain of each chamber was analyzed offline using two-dimensional STE software (Tomtec, REF-Version 4.6 software, Munich, Germany) by an experienced physician. LV GLS was assessed from apical 2-, 3- and 4-chamber views according to recommendations (14). RV GLS, RA, and LA longitudinal strains were assessed from apical 4C views (15).

### Statistical Analysis

Statistical analyses were performed using SPSS (version 17.0). Data were expressed as mean ± SD, and a test for normality was performed using the Kolmogorov–Smirnov test. The effects of hypoxic exposure on conventional echocardiographic parameters and cardiac mechanics were assessed by analysis of variance among the Han population and Tibetans. When a significant main effect was found, we used modified *t*-tests (i.e., *t*-tests with the residual variance of the analysis of variance) as a *post hoc* test to compare recent Han migrants with Han lowlanders and with long-term acclimatized Han migrants and to compare Native Tibetans with long-term Hans. *p*-values were adjusted for multiple comparisons using Bonferroni corrections. Statistical significance was declared when *p* < 0.05.

## Results

### Clinical Characteristics of the Subjects

Characteristics of the study population are summarized in Table 1. We found that Han migrants with 3–12 months of exposure to hypoxia had a moderately increased HR and BP. Elevations of HR and BP persisted in Hans during long-term (5–10 years) exposure. Also, hemoglobin concentrations and hematocrits continued to increase with persistently lower SpO_2_. Native Tibetans exhibited unique characteristics compared with to Han population, including relatively smaller BSAs and BMIs. They also had relatively lower BP, Hb, and hematocrits compared to highly acclimatized Hans.

**Table 1:** Clinic characteristics in Han lowlanders, Han migrants and native Tibetans.

	**Han lowlanders** **(*n* = 82)**	**Han migrants <1 year** **(*n* = 64)**	**Han migrants > 5 years** **(*n* = 71)**	**Tibetans** **(*n* = 75)**	***p***
Age (year)	25.3 ± 3.3	24.8 ± 3.3	26.3 ± 2.9	25.6 ± 4.2	0.086
Height (cm)	172.6 ± 5.2	173.4 ± 5.2	171.6 ± 6	171.9 ± 5.1	0.216
Weight (kg)	64.6 ± 4.9	66.4 ± 7.9	66.4 ± 7.9	62.4 ± 7^‡^	0.002
BSA (m^2^)	1.8 ± 0.1	1.83 ± 0.1	1.81 ± 0.1	1.77 ± 0.1^‡^	0.010
BMI (kg/m^2^)	21.7 ± 1.5	22.1 ± 2.2	22.5 ± 2.3	21.1 ± 2.1	0.001
HR (beats/min)	68.5 ± 6.1	80.2 ± 7.9*	78.8 ± 7^†^	73.5 ± 6.5^‡^	0.000
Systolic BP (mmHg)	113.3 ± 7.5	120.5 ± 6.4*	128.1 ± 8.5^†^	115.9 ± 8.4^‡^	0.000
Diastolic BP (mmHg)	70.3 ± 4.5	74.8 ± 4.6*	79.3 ± 5.2^†^	71.8 ± 5.8^‡^	0.001
Mean BP (mmHg)	84.6 ± 4.8	90 ± 4.4*	95.6 ± 5.6^†^	86.5 ± 5.7^‡^	0.000
Hb (g/l)	140.5 ± 10.6	175.5 ± 9.9*	180.5 ± 12.8^†^	173.7 ± 12.4^‡^	0.000
HCT (%)	46.3 ± 5.2	54.1 ± 3.9*	59.8 ± 4.9^†^	53.5 ± 3.9^‡^	0.000
SpO_2_ (%)	97.5 ± 1	90.5 ± 2.2*	90.9 ± 1.9^†^	89.2 ± 2^‡^	0.000

Values are means ± SD. *P <0.05 short-term acclimatized Hans vs. lowlanders; P < 0.05 long-term acclimatized Hans vs. short-term acclimatized Hans; P < 0.05 Tibetans vs. long-term acclimatized Hans.

*BSA, body surface area; BMI, body mass index; HR, heart rate; BP, blood pressure; Hb, hemoglobin; HCT, hematocrit; SpO_2_, arterial pulse oxygen saturation*.

### Right Heart Characteristics Under Chronic Hypoxia

**Figure 1:**
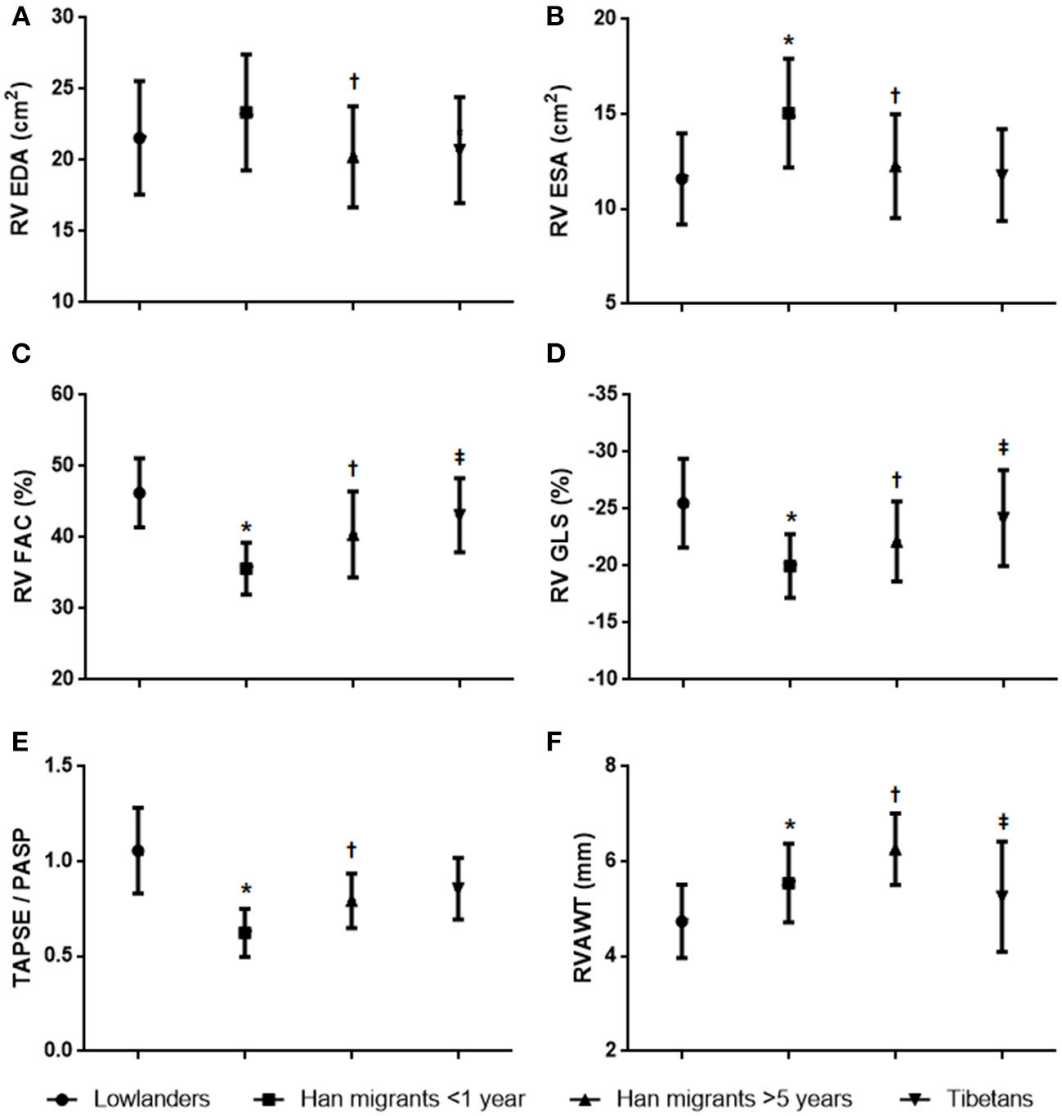
Between-group comparisons of right ventricular characteristics. **(A)** Right ventricular end-diastolic area. **(B)** Right ventricular end-systolic area. **(C)** Right ventricular fractional area change. **(D)** Right ventricular global longitudinal strain. **(E)** Tricuspid annular plane systolic excursion/pulmonary artery systolic pressure. **(F)** Right ventricular anterior wall sickness. *p < 0.05 Han migrants <1 year vs. lowlanders; †*p* < 0.05 Han migrants >5 years vs. Han migrants <1 year; ‡*p* < 0.05 Tibetans vs. Han migrants >5 years.

Figure 1 summarizes the main findings for right heart echocardiographic measurements; other echocardiographic parameters are provided in Table 2. Short-term acclimatized Hans exhibited enlarged RAs with preserved emptying fraction. RV FAC decreased significantly as a result of enlarged RV areas, especially the end-systolic area. Other parameters associated with RV systolic function such as se′ and TAPSE also decreased. Estimated PASP increased and the TAPSE/PASP ratio decreased significantly, indicating impaired RV-arterial coupling. As for cardiac mechanics, there were significant decreases in RV GLS with slightly decreased RA GLS.

**Table 2:** Echocardiographic parameters of right heart in Han lowlanders, Han migrants and native Tibetans.

	**Han lowlanders** **(*n* = 82)**	**Han migrants <1 year** **(*n* = 64)**	**Han migrants > 5 years** **(*n* = 71)**	**Tibetans** **(*n* = 75)**	***p***
RA EDVI (ml/m^2^)	15.6 ± 3.6	17.7 ± 5*	16.6 ± 4.4	18 ± 4.7	0.019
RA ESVI (ml/m^2^)	6.6 ± 1.9	7.7 ± 2*	7.5 ± 2.3	7.8 ± 1.9	0.010
RAEF (%)	57.7 ± 7.7	55.5 ± 9.6	55.2 ± 8.7	56.2 ± 7	0.415
RA GLS (%)	41.8 ± 8.8	38.5 ± 6.8*	40.4 ± 8.5^†^	41.1 ± 8.6	0.000
Tricuspid E (cm/s)	60.8 ± 8.2	56.6 ± 9.3*	50.9 ± 8.2^†^	63.9 ± 6.4	0.000
Tricuspid A (cm/s)	38.9 ± 6	33.5 ± 5.7*	40.1 ± 6.3^†^	39 ± 5	0.000
Tricuspid E/A ratio	1.6 ± 0.2	1.7 ± 0.3	1.3 ± 0.2^†^	1.6 ± 0.2^‡^	0.000
s′ (cm/s)	12.5 ± 1.7	11.7 ± 1.9*	11.9 ± 1.5	12.7 ± 1.8^‡^	0.001
e′ (cm/s)	14 ± 2	13.2 ± 2.9*	12.6 ± 2.5	14.1 ± 2.1^‡^	0.000
a′ (cm/s)	12.2 ± 1.9	9.2 ± 1.8*	11.4 ± 1.2^†^	11.6 ± 2	0.000
Tricuspid E/e' ratio	4.4 ± 0.9	4.4 ± 1	4.2 ± 1	4.6 ± 0.8^‡^	0.034
TAPSE (mm)	21.6 ± 1.6	17.8 ± 3*	20.8 ± 3^†^	21.3 ± 2.1	0.000
P_TR_ (mmHg)	17.9 ± 3	27.5 ± 4.2*	23.6 ± 3.7^†^	22.9 ± 2.8	0.000
RAP (mmHg)	3.2 ± 1.1	4.4 ± 2.2*	3.6 ± 1.6^†^	3.4 ± 1.4	0.000
PASP (mmHg)	21.1 ± 4.2	32.6 ± 5.1*	27.8 ± 5.2^†^	26.1 ± 3.4	0.000
PAMP (mmHg)	15.4 ± 3.8	22.6 ± 4.6*	19.2 ± 4.7^†^	18.26 ± 3.8	0.000

Values are means ± SD. *P < 0.05 short-term acclimatized Hans vs. lowlanders; P < 0.05 long-term acclimatized Hans vs. short-term acclimatized Hans; P < 0.05 Tibetans vs. long-term acclimatized Hans.

*EDVI, end-diastolic volume index; ESVI, end-systolic volume index; RAEF, right atrial emptying fraction; GLS, global longitudinal strain; TAPSE, tricuspid annular plane systolic excursion; P_TR_, tricuspid regurgitation gradient pressure; PASP, pulmonary artery systolic pressure; RAP, right atrial pressure; PAMP, pulmonary artery mean pressure*.

Compared with short-term Han migrants (3–12 months), RV FAC and TAPSE improved during a longer period of acclimatization (>5 years), and changes were also accompanied by reductions of RV end-diastolic and end-systolic area, almost back to the normal values as lowlanders. There was also evidence of RV anterior wall thickening. Importantly, RV GLS of longer migrants was also higher with improvements in RV-PA coupling (i.e., improved values of TAPSE/PASP).

Tibetans had higher RV FACs without thickening of the RV wall compared with long-term Han migrants. PASP in Tibetans was lower than in Han migrants and showed better RV-arterial coupling. Notably, Tibetans had nearly unchanged RV GLS compared to lowlanders, demonstrating more favorable adaptation to chronic hypoxia than highly acclimatized Han migrants.

### Left Heart Characteristics Under Chronic Hypoxia

**Figure 2:**
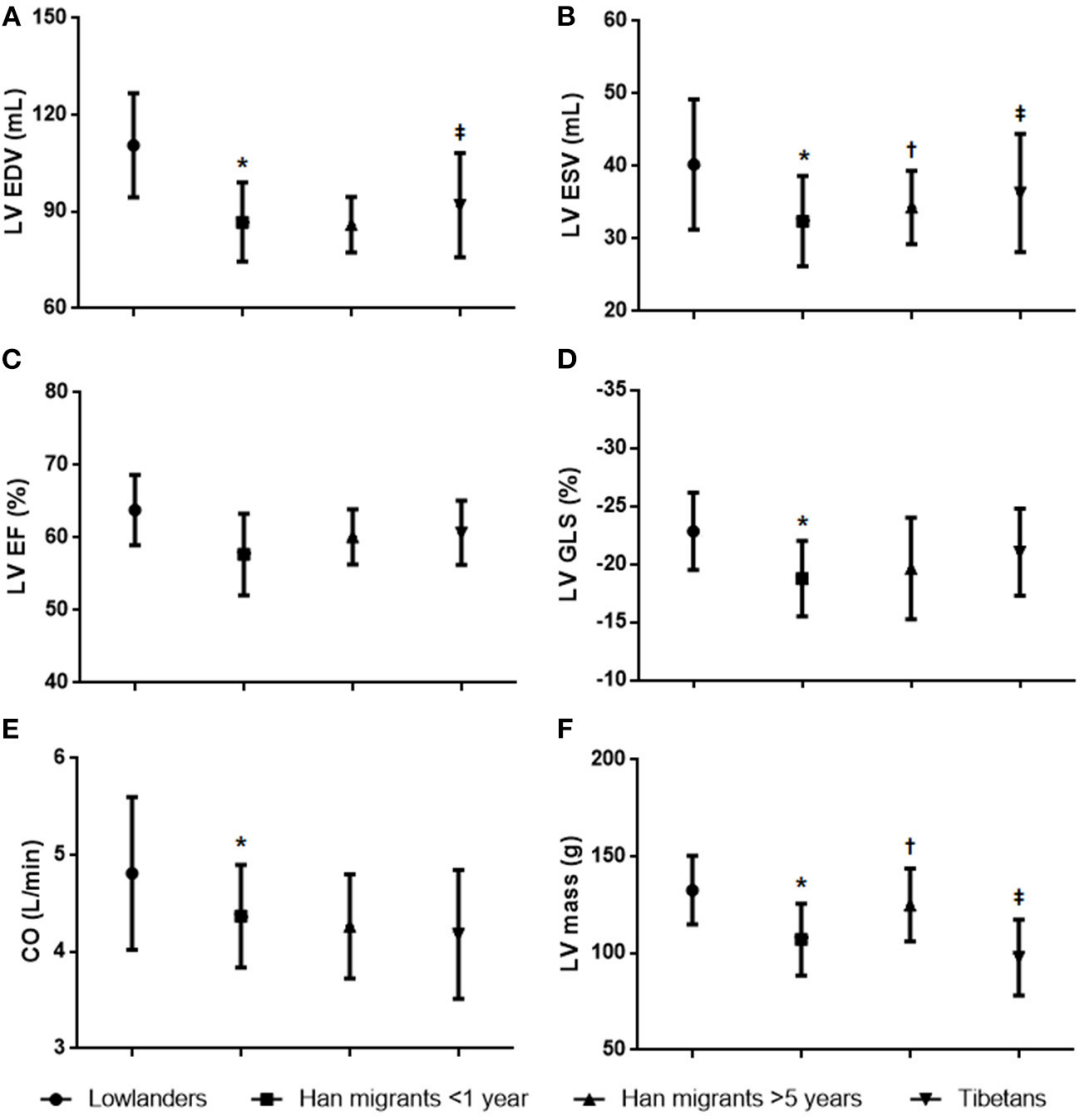
Between-group comparisons of left ventricular characteristics. **(A)** Left ventricular end-diastolic volume. **(B)** Left ventricular end-systolic volume. **(C)** Left ventricular ejection fraction. **(D)** Left ventricular global longitudinal strain. **(E)** Cardiac output. **(F)** Left ventricular mass. *p < 0.05 Han migrants <1 year vs. lowlanders; *p* < 0.05 Han migrants >5 years vs. Han migrants <1 year; ‡*p* < 0.05 Tibetans vs. Han migrants >5 years.

Left heart echocardiographic parameters are summarized in Figure 2 and Table 3. Significant changes in left heart structure under short-term exposure were characterized by reduced volumes of both LA and LV. LVEF was preserved, and there was a significant decrease in peak E and a mildly decreased E/e′. IVRT increased slightly with enhanced DT. As for cardiac mechanics, LV GLS was decreased, whereas LA GLS was preserved. Overall, these findings suggest that short-term exposure to chronic hypoxia resulted in decreased LV fillings but preserved systolic and diastolic functions.

**Table 3:** Echocardiographic parameters of left heart in Han lowlanders, Han migrants and native Tibetans.

	**Han lowlanders** **(*n* = 82)**	**Han migrants <1 year** **(*n* = 64)**	**Han migrants > 5 years** **(*n* = 71)**	**Tibetans** **(*n* = 75)**	***p***
**Conventional and Doppler data**					
LA EDVI (ml/m^2^)	20.3 ± 4.4	16.9 ± 3.3*	17.1 ± 3	17.8 ± 3.8	0.000
LA ESVI (ml/m^2^)	7 ± 1.6	6.7 ± 1.8	6.6 ± 1.8	7.6 ± 1.9^‡^	0.017
LAEF (%)	65.3 ± 5.3	60.3 ± 6.9*	61.7 ± 7.7	57.5 ± 6.2^‡^	0.000
LA GLS (%)	42.6 ± 7.1	40.2 ± 4.7	40.5 ± 6.9	38.8 ± 6.6	0.000
IVST (mm)	8.5 ± 1.1	8.7 ± 1.4	9.5 ± 1.1^†^	7 ± 1.1^‡^	0.000
LVDD (mm)	49.7 ± 4.8	41.7 ± 3.1*	42.5 ± 2.3	46.1 ± 3.6^‡^	0.000
LVPWT (mm)	7.3 ± 0.8	8.1 ± 1.4*	8.8 ± 1^†^	6.7 ± 1.1^‡^	0.000
Mitral E (cm/s)	85.2 ± 10.8	64.2 ± 11.2*	59.8 ± 9.2^†^	77.9 ± 10.3^‡^	0.000
Mitral A (cm/s)	45.2 ± 6.5	50.1 ± 7.1*	46.9 ± 8.1^†^	48.4 ± 9.2	0.000
Mitral E/A ratio	1.8 ± 0.2	1.6 ± 0.3	1.3 ± 0.2^†^	1.7 ± 0.2^‡^	0.000
e′ (cm/s)	17.6 ± 2	16 ± 2.8*	14.3 ± 2^†^	18.5 ± 1.8^‡^	0.000
a′ (cm/s)	7.6 ± 1.4	8.1 ± 1.6	9 ± 1.8^†^	7 ± 1^‡^	0.000
Mitral E/e′ ratio	5.2 ± 1.3	4.1 ± 1*	4.2 ± 0.7	4.8 ± 1.3^‡^	0.000
IVRT (ms)	62.9 ± 5	73.2 ± 6.8*	80.8 ± 7.4^†^	82 ± 8.8	0.000
DT (ms)	171.3 ± 26.5	177.6 ± 23.8*	189 ± 21.4^†^	157.7 ± 14.2^‡^	0.000

Values are means ± SD. *P < 0.05 short-term acclimatized Hans vs. lowlanders; P < 0.05 long-term acclimatized Hans vs. short-term acclimatized Hans; P < 0.05 Tibetans vs. long-term acclimatized Hans.

*EDVI, end-diastolic volume index; EDVI, end-systolic volume index; LAEF, left atrial emptying fraction; GLS, global longitudinal strain; IVST, interventricular septal thickness LVDD, left ventricular end diastolic diameter; LVPWT, left ventricular posterior wall thickness; IVRT, isovolumic relaxation time; DT, deceleration time*.

Compared with short-term Han migrants, LA and LV volumes of longer migrants (>5 years) remained small, and LVEF was also preserved. Notably, LV mass index was higher due to increased interventricular septum and posterior wall thicknesses. Mitral E/A and E/e′ remained low. IVRT and DT increased with preserved LA and LV GLS. This suggested that longer exposure to hypoxia caused significant LV thickening but no evidence of diastolic dysfunction or depression of cardiac mechanics.

In contrast, Tibetans had relatively larger LAs and LVs compared to highly acclimatized Hans, but no differences were noted in LVEF. Also, the LV mass index was smaller in Tibetans as they had no significant LV thickening. Additionally, LV GLS was also maintained within a normal range. Thus, overall, Tibetans demonstrated a more highly adapted pattern of cardiac characteristics than long-term acclimatized Han migrants.

## Discussion

**Figure 3:**
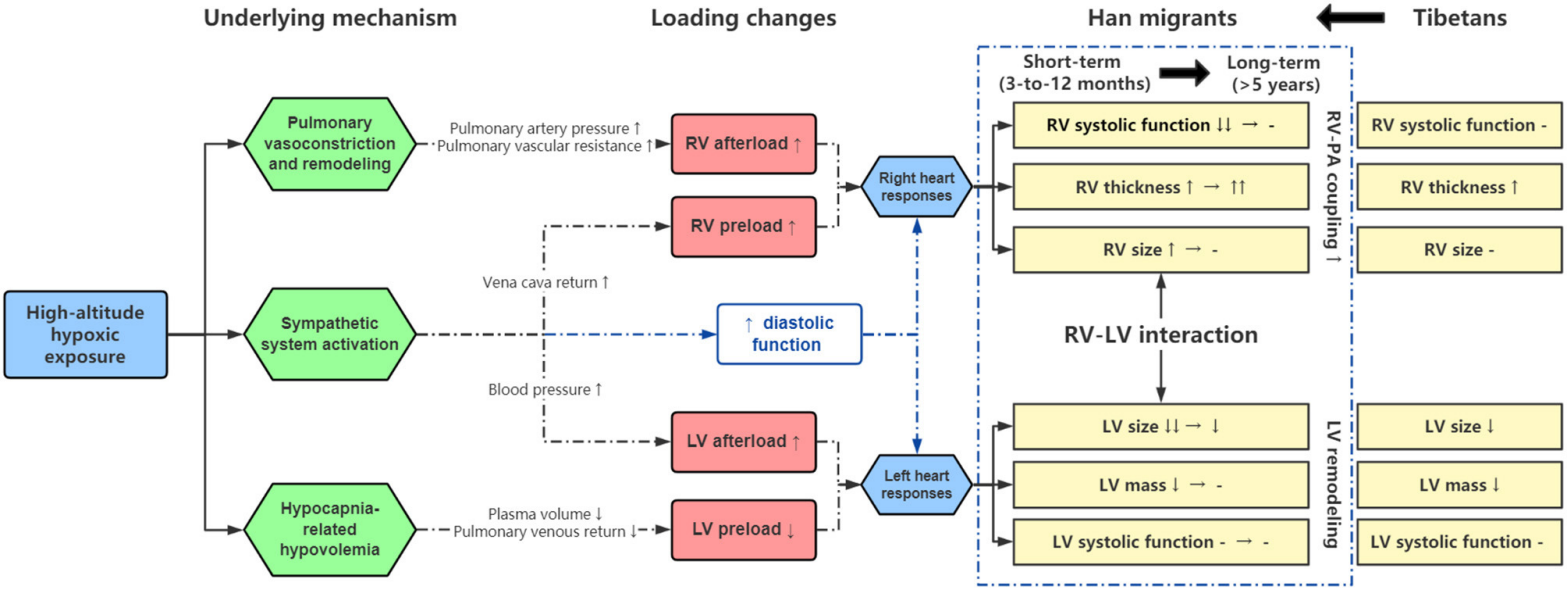
Summary of cardiac adaptations to prolonged exposure to chronic hypoxia. Refer to text for details.

Although previous studies evaluated cardiovascular alterations in response to acute hypoxia at high altitudes, this study is the first to investigate the effects of different stages of chronic exposure on biventricular structure, function, and mechanics. By comparing clinic and echocardiographic measures among recent (3–12 months) Han migrants, long-term (>5 years) Han migrants, and native Tibetans, we observed that (1) recent Han migrants showed a significant response to hypoxia that was characterized by increased RV size, decreased RV FAC, and decreased longitudinal strain of both ventricles; (2) all of these factors improved during long-term (>5 years) dwelling, and while there was improved right ventricular-pulmonary arterial coupling, there was persistent ventricular thickening; (3) even after long-term exposure, significant differences in cardiac structure, function, and mechanics existed compared to the more highly adapted native Tibetans. The major findings of this study, along with proposed links between high altitude dwelling, cardiac structure and function, are summarized in Figure 3 and are discussed further below.

### RV Systolic Function Is Impaired in Recent (<1 Year) Migrants to High Altitudes but Improved During Long-Term (>5 Years) Dwelling

One of the important findings of our study was the adaptability of the RV and pulmonary vasculature of Han migrants to prolonged high altitude dwelling. Prior studies have shown that elevated PASP is the most immediate and prominent hemodynamic response to high altitude (16, 17). A recent survey showed that high altitude pulmonary hypertension (HAPH) could be detected in 6–35% of highlanders in the Asian population (18). Specifically, we observed a PASP of 33 ± 5 mmHg in recently adapted Han migrants, which is at the normal upper limit of healthy lowlanders; furthermore, 10% of Han migrants fulfilled the 2016 ERS/ESC guideline criteria for pulmonary hypertension (PH) with a PAMP >25 mmHg (19). Despite the increased afterload and noted changes of right ventricular properties measured in Han migrants (i.e., decreased RV FAC, TAPSE, and GLS), subjects were asymptomatic, and according to a recent expert consensus document, such responses may not have clinical significance. Specifically, it was noted that in high-altitude dwellers, the criteria for defining HAPH were PAMP > 30 mmHg and PASP > 50 mmHg in asymptomatic high-altitude dwellers (20); none of the subjects studied met these criteria. As reported previously, normal RV contractile reserve was observed in healthy high-altitude dwellers and even in patients with chronic mountain sickness (CMS), despite lower resting values of RV function (21). Also, a prior study showed that acute HA exposure impairs RA function in normal individuals (22), which has been shown to be a sensitive and valuable metric in assessing RV reserve and predicting exercise capacity (23, 24). In contrast, our study demonstrated that during prolonged adaption to high altitude, there was no significant depression of RA emptying fraction or RA GLS in Han migrants. This indicates that chronic exposure to hypoxia may not prevent adequate adaption to exercise of these healthy migrants despite decreased RV systolic function at rest.

Interestingly, the RV demonstrated significant adaptability, as we observed near normalization of RV size and functions in migrants with more than 5 years of adaption. The reasons for improvement may be partly explained by the concomitant decreases in PASP, increase in pulmonary oxygenation, and improved RV-PA coupling. Based on prior literature, mechanisms contributing to these improvements may include hypoxia-related activation of sympathetic tone, increased ventilation, and hypocapnia-induced hypovolemia (25). Evidence for increased sympathetic tone was provided by our observation that Hans always maintained a faster heart rate and smaller stroke volume during long-term high altitude dwelling. Furthermore, several studies demonstrated that acute hypoxia and HA exposure lead to a decline in heart rate variability (HRV), which indicates a marked increase in sympathetic tone (26, 27).

### LV Remodeling Persists in Long-Term (>5 Years) Migrants but Was Not Associated With Impairment of LV Systolic or Diastolic Function

**Figure 4:**
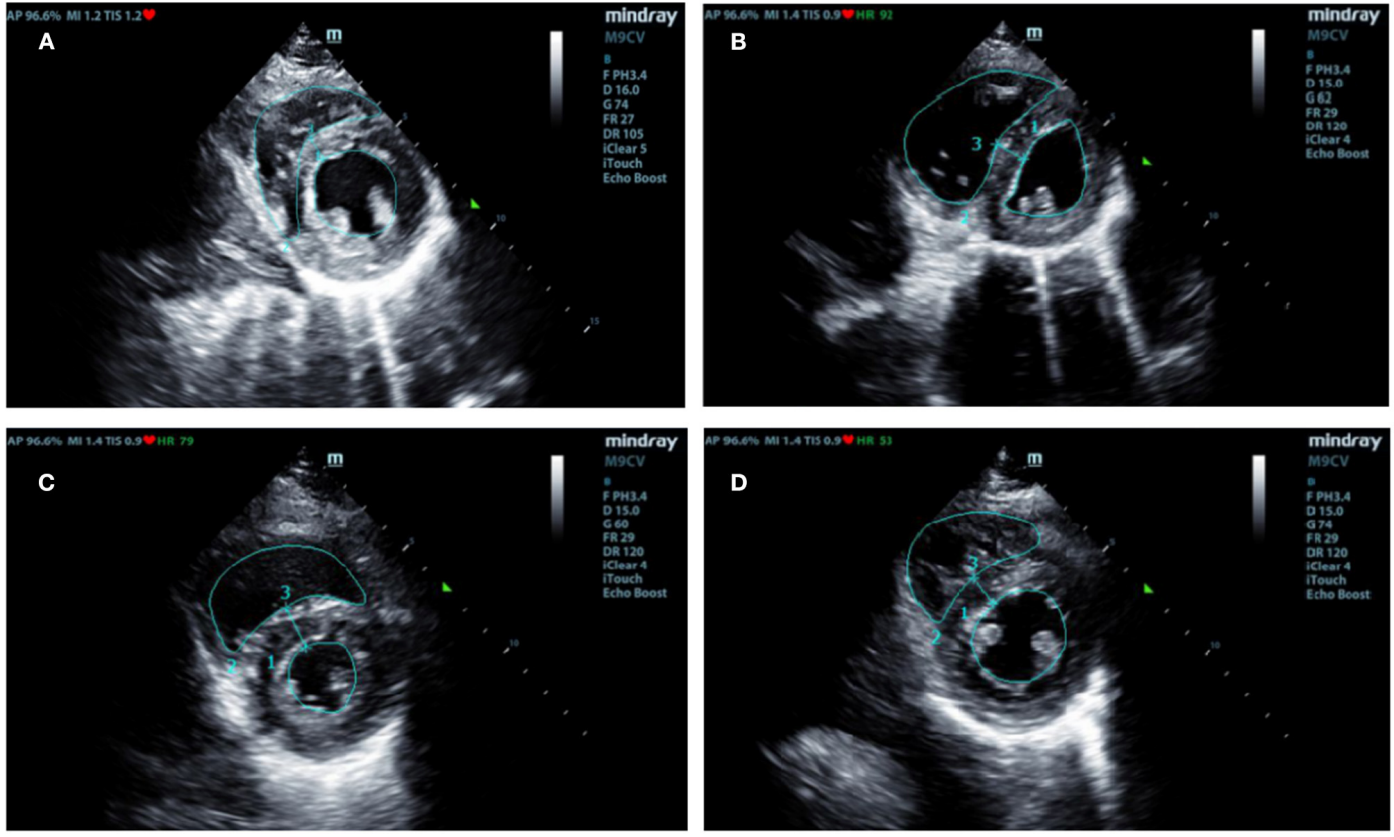
Typical changes of cardiac morphology during various durations of exposure to high altitude. End-systolic short-axis images. **(A)** Han lowlanders (controls) with normal volume and thickness. **(B)** Short-term Hans migrants (<1 year) had enlarged RV with the leftward shifted interventricular septum. **(C)** Long-term Hans migrants (>5 years) had significantly thickened LV wall with recovered RV size. **(D)** Native Tibetans had no or minimal differences in LV structure compared with Han lowlanders. RV, right ventricular; LV, left ventricular.

Han migrants showed significant LV remodeling characterized by decreased chamber size and increased wall thickness during the high-altitude dwelling. RV–LV interactions may have played an important role in the altered LV structure during 3–12 time frames. As shown in Figures 4A,B, the LV size was visibly reduced due to the leftward displacement of the ventricular septum. Moreover, decreased LV GLS was also accompanied by the decreased RV GLS we observed in recent Han migrants. However, the LVEF (overall systolic function) was preserved.

Moreover, despite the RV structure of Han migrants improving nearly back to normal during >5 years of adaption, the LV remained small with the increasingly thickened wall (Figure 4C). The small and thickened LV in long-term Han migrants may be due to persistent under-filling conditions and increased afterload as indexed by the persistently decreased mitral E/A and E/e′ and increased blood pressure, respectively. Several factors may contribute to such conditions. On the one hand, an increase in PASP reduced blood return to the left atrium. On the other hand, hypoxia and hypothermia at high altitude activated the sympathetic nervous system, which resulted in compensatory increases in ventilation, heat production, and peripheral vasoconstriction (28). In turn, these may lead to hypocapnia-related hypovolemia, an increase in LV afterload, and persistently increased LV wall thickness during the prolonged high-altitude dwelling.

Notably, such LV remodeling may not cause a significant decrease in diastolic function in these young healthy migrants. Previous studies reported that age was the most significant risk factor for the occurrence of left ventricular diastolic dysfunction among high-altitude dwellers (29). However, no evidence of diastolic dysfunction was observed in our subjects according to the definition of recent guidelines (30). We only observed slight changes in early myocardium relaxation (i.e., increased IVRT and DT) but without increased LV filling pressure (i.e., normal E/e′). In this regard, age should be taken into account as the subjects we enrolled were almost young migrants who may not be prone to develop LV diastolic dysfunction. Furthermore, as noted above, significant increases in sympathetic tone may contribute to preserving LV diastolic functions.

### Tibetans Show Different Patterns of Adaptation to Chronic Hypoxia Compared With Highly Acclimatized Hans

Another finding of our study was that cardiac properties of highly acclimatized Hans do not fully return to normal. In contrast, native Tibetans show minimal differences in cardiac properties compared to lowlanders. In terms of cardiovascular properties, Tibetans have minimally altered cardiac structure, function, or mechanics. First, Tibetans showed a slight increase in PASP and a more favorable RV-PA coupling which did not cause a significant decrease in RV functions compared to Han migrants. In addition, relatively lower HR and BP in Tibetans suggest lower sympathetic tone and, therefore, would be less prone to LV thickening during long-term hypoxia exposure (Figure 4D). In terms of physiology, it was known that hypoxia can stimulate the increase of erythropoietin (EPO) and increase the number of red blood cells and hemoglobin concentration to maintain the normal arterial oxygen content (31, 32). Notably, lowlander migrants showed persistently increased hemoglobin and hematocrit along with prolonged dwellings at high altitudes. However, the excessive increase of hematocrit (> 50%), we observed, in most long-term migrants (>5 years) may increase blood viscosity, slow blood flow, and increase cardiac afterload, which can accelerate cardiac remodeling and increase the risk to develop premature cardiovascular disease (33). In contrast, there was a moderate increase of Hb and hematocrit in Tibetans that may not lead to the significant side effects of increased blood viscosity. Such a more favorable pattern of adaptation may reflect the long-term process of natural selection and generational evolution (34). Genetic differences between the Han population and Tibetans may provide important clues to mechanisms of cardiac and physiological adaption to high altitude, which should be given further consideration.

## Limitations

This study has several limitations. First, this was a cross-sectional study comparing changes in cardiovascular properties in response to hypoxia in short vs. long-term migrants and long-term Hans vs. Tibetans; this cannot rule out the impact of individual variability on the results compared with a longitudinal study. Second, we did not analyze subjects of migrants with 1–5 years of adaptation due to the small number of subjects falling within this timeframe. Third, we did not enroll patients with high altitude pulmonary edema (HAPE) or CMS whose cardiovascular features have been reported in previous studies. In this regard, the results of this study provide important information concerning cardiovascular adaptations of healthy migrants to high altitudes.

## Conclusion

Han migrants show improved RV structure and function during the long-term dwelling. However, LV remodeling persists without impairment of LV systolic or diastolic function. Despite after >5 years of acclimatization, cardiac properties of Han migrants do not fully return to normal. In contrast, native Tibetans show minimal differences in cardiac properties compared to lowlanders.

## Data Availability Statement

The original contributions presented in the study are included in the article/supplementary material, further inquiries can be directed to the corresponding author/s.

## Ethics Statement

The studies involving human participants were reviewed and approved by Chinese PLA General Hospital Ethics Committee. The patients/participants provided their written informed consent to participate in this study.

## Author Contributions

XC, DB, and KH contributed to conception and design of the study. BL, QJ, and WC organized the database. FY and WW performed the statistical analysis. XC wrote the first draft of the manuscript. LY, ZL, QZ, and YL wrote sections of the manuscript. PZ and HP made charts and figures. All authors contributed to manuscript revision, read, and approved the submitted version.

## Funding

This study was supported by the Science and Technology Innovation Special Zone: No. 19-163-12-ZD-037-003-02.

## Conflict of Interest

HP and PZ were employed by BioMind Technology Company. The remaining authors declare that the research was conducted in the absence of any commercial or financial relationships that could be construed as a potential conflict of interest.

## Publisher's Note

All claims expressed in this article are solely those of the authors and do not necessarily represent those of their affiliated organizations, or those of the publisher, the editors and the reviewers. Any product that may be evaluated in this article, or claim that may be made by its manufacturer, is not guaranteed or endorsed by the publisher.
